# Memantine in Psychiatry: An Underutilized Therapeutic Breakthrough?

**DOI:** 10.1192/bjo.2025.10795

**Published:** 2025-06-20

**Authors:** Harsimar Kaur, Saksham Sharma

**Affiliations:** 1 ANR Neuropsychiatry Centre, Jalandhar, India; 2 University of Nis, Nis, Serbia

## Abstract

**Aims:** Memantine, an N-methyl-D-aspartate receptor antagonist, is primarily approved for the treatment of Alzheimer’s disease and other forms of dementia. However, multiple studies have explored its potential applications in psychiatric disorders. This literature review aims to evaluate existing evidence on the efficacy of memantine in conditions such as trichotillomania, skin picking disorder, depression, bipolar disorder, obsessive-compulsive disorder, ADHD, autism spectrum disorder, substance use disorder, schizophrenia, and reducing cognitive effects of electroconvulsive therapy. The review hypothesizes that, based on available literature, memantine may have therapeutic potential across these psychiatric disorders, particularly as an adjunct treatment.

**Methods:** A literature search was conducted using PubMed from 2020 to 2025 (5 years), employing the search strategy ((Memantine) AND (Psychiatry)) NOT (Dementia). This literature review was conducted by screening 27 searched titles. The inclusion criteria encompassed systematic reviews, meta-analysis and randomised controlled trials. Exclusion criteria included animal and cell studies, case studies, reviews, editorials, case reports, and letters to editors. A total of 23 papers were included in this review and 4 were excluded as they focused on conditions outside the scope of this review.

**Results:** Emerging evidence from the 23 selected studies (9 RCTs, 8 systematic reviews, 2 meta-analyses, and 4 combined systematic reviews with meta-analyses) suggests that memantine may be beneficial across various psychiatric disorders, particularly as an adjunct. Most of the available literature points towards a positive response of using memantine in conditions like trichotillomania, skin picking disorder, depression, bipolar disorder, obsessive-compulsive disorder, ADHD, autism spectrum disorder, substance use disorder, schizophrenia, mitigating cognitive effects of electroconvulsive therapy, and other conditions. However, the number of studies per disorder that met the inclusion criteria was limited. This highlights the lack of extensive research for individual disorders and the need for further clinical validation.

**Conclusion:** Memantine’s NMDA receptor antagonism offers a promising yet underexplored therapeutic approach in psychiatry. While preliminary findings suggest potential benefits across multiple psychiatric disorders, the number of high-quality studies per condition remains very limited, with most disorders represented by only three or four studies meeting the inclusion criteria. This underscores the need for more randomized controlled trials with larger sample sizes to validate its efficacy, refine dosing strategies, and assess long-term safety. Expanding research in this area is crucial to clarify memantine’s role in psychiatric practice and prevent its underutilization.

